# The role of hospital pharmacists in supporting the appropriate and safe use of CGT/ATMPs: a scoping review of current insights

**DOI:** 10.1186/s12913-024-12026-4

**Published:** 2025-01-09

**Authors:** Junnan Shi, Xianwen Chen, Hao Hu, Carolina Oi Lam Ung

**Affiliations:** 1 State Key Laboratory of Quality Research in Chinese Medicine, Institute of Chinese Medical Sciences, University of Macau, Macao SAR, China; 2 Centre for Pharmaceutical Regulatory Sciences, University of Macau, Macao SAR, China; 3 Department of Public Health and Medicinal Administration, Faculty of Health Sciences, University of Macau, Macao SAR, China

**Keywords:** Hospital pharmacy, Pharmaceutical care, Pharmacy practice, Advanced therapy medicinal products, Cell and gene therapy, Regeneration medicine

## Abstract

**Background:**

The role of hospital pharmacists in managing cell and gene therapy (CGT) and advanced therapy medicinal products (ATMPs) is gradually being recognized but the evidence about impact of their role has not been systematically reported.

**Objective:**

This study was aimed to summarize the professional services provided by hospital pharmacists on managing CGT/ATMPs and the evidence about the effects on patient care, as well as to identify the perceptions about pharmacists assuming a role that supports the appropriate and safe use of CGT/ATMPs.

**Methods:**

Literature from 4 electronic databases (PubMed, ScienceDirect, Web of Science, Scopus) were searched following PRISMA checklist to yield publications on the interventions provided by hospital pharmacists in the management of CGT/ATMPs dated since 1 January 2013 till 30 April 2023.

**Results:**

Thirty-four publications were included in this review. Eight studies involving hospital pharmacists participating in interventions for 1,012 hematopoietic stem cell transplant (HSCT) patients from 8 hospitals in 5 countries were identified. Common pharmacist-led interventions centered on medicine administration, prescribing, and monitoring of medicines use, resulting in significant improvement in patient adherence, satisfaction and knowledge. Of 26 studies, the perspectives assuming their roles in CGT/ATMPs management were categorized when patients receiving ATMPs (*n* = 2), HSCT and cellular-based therapy (*n* = 12), gene therapy (*n* = 6), and the chimeric antigen receptor (CAR) T-cell therapy (*n* = 6), mainly covering procurement, influences on prescribing, preparation and delivery, administration, monitoring of medicines use, human resources, training and development. The anticipated impact was primarily intended to promote pharmacy practice, multidisciplinary collaboration and improve patient clinical outcomes.

**Conclusion:**

Leveraging the role of hospital pharmacists in multidisciplinary healthcare teams to develop a coordinated approach that supports pharmacy practice will better meet the management of CGT/ATMPs. For hospital pharmacists to step up their role in the multidisciplinary healthcare team, advancing their skillset in terms of clinical practice standards and medication management is essential.

**Supplementary Information:**

The online version contains supplementary material available at 10.1186/s12913-024-12026-4.

## Introduction

**Fig. 1:**
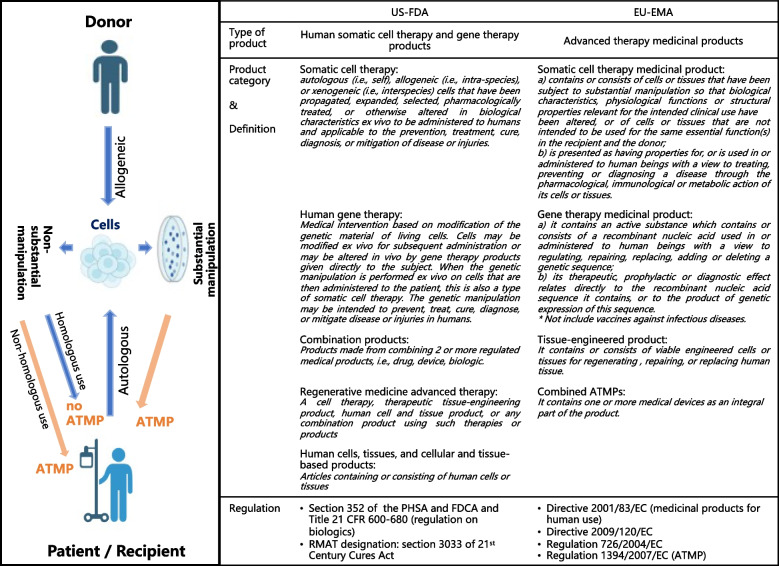
The definition of CGT/ATMPs in the US and the EU [3, 4]. (^*^The Directive 2001/83/EC and Regulation 1394/2007/EC, introduced the concept of two different types of therapeutic ceels (‘substantially modified’ versus ‘minimally manipulated’ or ‘non-substantially modified’). ‘Minimally manipulated’ or ‘non-substantially modified’ cells and tissues are not medicines but are CGT, such as the conventional haematopoietic stem cell transplant (HSCT).)

Novel cell and gene therapies (CGT) offer groundbreaking opportunities for the treatment of diseases and injuries by leveraging modified nucleic acids, altered cells or tissue, or both [1]. The European Union (EU) refers to these therapies as advanced therapy medicinal products (ATMPs) and categorizes them into four subtypes: gene therapy medicines, somatic-cell therapy medicines, tissue- engineered medicines, and combined ATMPs [2]. The terms “CGT”, “ATMPs” and “regeneration medicines” are often used interchangeably. According to the definition provided by the United States (US) and the EU, there are regulatory discrepancies between the 2 systems in terms of definitions and sub-classifications show some regulatory (Fig. 1) [3, 4]. In this review, both the terms CGT and ATMPs are included within the study scope to ensure the comprehensiveness of the findings.

CGT/ATMPs hold great potential for reshaping the progression or the disability associated with multiple diseases such as Alzheimer's disease, Parkinson's disease, cancer, muscular dystrophy, and so on [5, 6]. These therapies offer the possibility of curing or reversing diseases that are currently untreatable or only subject to symptomatic relief. For example, Chimeric antigen receptor (CAR) T-cell therapy has shown remarkable performance in treating blood cancers, and its advances, in combination with other therapeutic approaches, have opened vast prospects for more effective cancer treatments [7].

However, the clinical use of CGT/ATMPs faces specific challenges due to the complex nature of these medical products and the limited availability of clinical data [8]. These challenges include the need for procurement capability, integrated logistics solutions, skilled staff, fully traceable supply chains, specialized on-site freezing and thawing equipment and expertise, data infrastructure for long-term patient follow-up, and streamlined health economic and procurement models [9]. The successful clinical adoption of CGT/ATMPs also largely depends on the medical team’s ability to perform these services [10]. In the absence of extensive experience, the high-quality and safe harnessing of the complex biological mechanisms of CGT/ATMPs requires collaboration among a multidisciplinary medical team in the clinical setting.

As an indispensable member of the hospital healthcare team, pharmacists play a vital role in the efficient management of various diseases, such as cancer [11], genetic disorders [12], autoimmune disorders [13], rare diseases [14], which are common treatment areas for patients receiving CGT/ATMPs [15, 16]. With the rapid increase in the number of registered clinical trials and market-authorized products worldwide, the adoption of ATMPs to alleviate or treat diseases in the clinical setting is becoming more common [17]. Marzal et al. [18] reported the critical role of pharmacists in ensuring the safe and reliable use of CAR T-cell drugs, including assisting in the preparation of drugs, managing shipment and storage, and being involved in patient evaluation, patient education, pharmacovigilance and monitoring. The role of hospital pharmacists in the management of CGT/ATMPs is gradually being recognized [18], but the evidence on it and the impact on patient outcomes has not been systematically reported. The objective of this study was to identify the empirical evidence of pharmacist interventions for patient receiving CGT/ATMPs, and to summarize the perceptions and opinions about pharmacists assuming a role that supports the appropriate and safe use of CGT/ATMPs.

## Methods

This scoping review was conducted and reported according to the Preferred Reporting Items for Systematic reviews and Meta-Analyses extension for Scoping Reviews (PRISMA-ScR) checklist and utilized the Basel Statements to classify the types of hospital pharmacists’ interventions [19, 20]. The review protocol was registered with PROSPERO (CRD42023424699) and can be accessed at https://www.crd.york.ac.uk/prospero/display_record.php?RecordID=424699.

### Search strategy

The search was conducted across four databases (PubMed, Scopus, Web of Science, and Science Direct) for peer-reviewed articles published from January 1, 2013, to April 30, 2023 that reflect the hospital pharmacists’ role in the management of contemporary advanced therapies and ATMPs. The two primary search terms were “pharmacist” (“pharmacy” OR “pharmacist*” OR “pharmaceutical service*” OR “pharmaceutical care”) and “CGT/ATMPs” (“cell*” OR “gene” OR “tissue” OR “advanced therap*” OR “biological therap*” OR “regenerative therap*” OR “regenerative medicine*”). Medical Subject Headings (MeSH) terms were added to ensure a comprehensive search strategy, including “Pharmacy [MeSH]”, “Pharmacists [MeSH]”, “Pharmaceutical Services [MeSH]”, “Biological Therapy [MeSH]” and “Regenerative Medicine [MeSH]”. Terms within “pharmacist” and “CGT/ATMPs” were combined with OR, and the results from each concept were combined with AND. The reference lists and citations of included articles were also assessed for further articles that met the inclusion criteria.

### Inclusion/exclusion criteria

Full text peer-reviewed articles published in English that discussed the roles, responsibilities, and competencies of hospital pharmacists in CGT/ATMPs were eligible for inclusion. Articles involving related opinions and recommendations for hospital pharmacists in the adoption and management of CGT/ATMPs were also included in this review. No further limitations were applied to the initial search to ensure all relevant articles were captured. All study designs including observational studies (case report, case series, cross-sectional, case–control, cohort studies), studies involving an intervention (quasi-experimental studies, randomized controlled trials, community trials, field trials), reviews and commentaries were considered. Conference presentations were not eligible to be included in this review.

### Study selection

PRISMA-ScR checklist was used to screen the related literature. After removing the duplicate articles (JS), the titles and abstracts identified in the search were independently screened by two authors (JS and XC). The remaining articles were screened for inclusion based on full text separately by JS, XC and COLU. To ensure the quality of the screening process, the screening results were compared between JS and XC. Any conflicts in findings were discussed and resolved between JS and XC and confirmed by a third author (COLU) to reach agreement. To further ensure consistency in the application of inclusion/exclusion criteria, studies recommended for exclusion were finalized based on consensus by all authors.

### Data collection and analysis

Relevant data involving pharmacists’ interventions in practical hospital setting were extracted from the included articles into an Excel table. The extracted data included the name of the first author, year of publication, paper type/study design, type of subjects, study content (including study location, number of hospitals, number of patients, duration of intervention, if applicable), study objectives, interventions, major outputs and outcomes, and conclusions or recommendations. In addition, another standardized extraction form was used to extract data from eligible articles on views, opinions, recommendations regarding hospital pharmacists’ roles, responsibilities, competencies or any other relevant description in managing CGT/ATMPs. This includes details on the types of therapies, study content, key findings, and anticipated outcomes or impacts.

The types of interventions were classified according to the 2015 version of the Basel Statements by the Global Conference on the Hospital Pharmacy Section of the International Pharmaceutical Federation (FIP) [19]. These statements cover all areas of the medicine use process in hospitals, including: (1) Procurement, (2) Influences on Prescribing, (3) Preparation and Delivery, (4) Administration, (5) Monitoring of Medicines Use, and (6) Human Resources, Training and Development. The category “Others” was added to accommodate any interventions beyond the 6 areas described in the Basel Statements. Patient outcomes were defined according to the economic, clinical, and humanistic outcome (ECHO) model and were grouped into economic outcomes, clinical outcomes, and/or humanistic outcomes [21, 22].

The content analysis approach was applied to group data into common concepts and categorize findings based on the Basel Statements and the ECHO model. JS and XC extracted the data through a full-text review for further analysis. To ensure the consistency of data extraction, data extraction for the first five articles was performed separately by COLU and the two authors (JS and XC) to compare and assess the accuracy and consistency of the extracted data. For quality assurance purposes, COLU and HH cross-checked the analysis results to ensure the accuracy and completeness of the extracted data. No assessments of publication quality were made to evaluate the potential bias or systematic error as the current study was not designed to investigate the effect of an intervention or exposure.

## Results

### Study selection

**Fig. 2:**
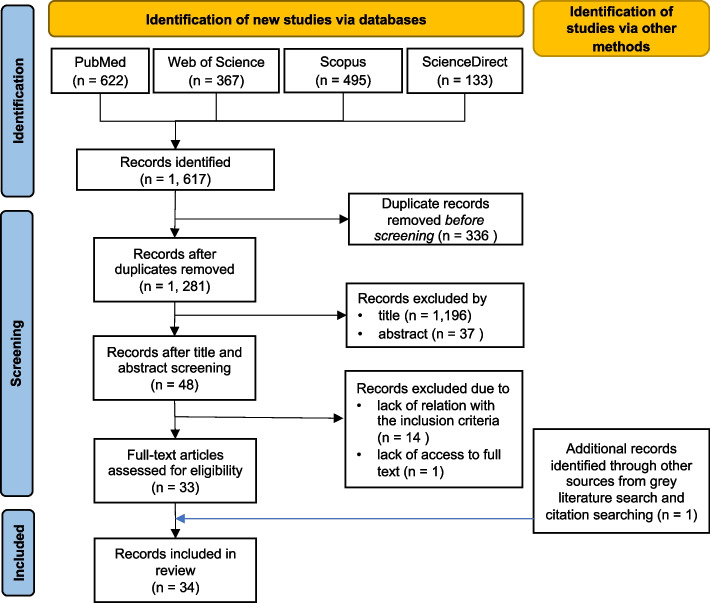
Flowchart of study selection criteria following the PRISMA-ScR checklist

The initial search identified 1,617 articles, of which 336 duplicates were removed resulting in 1,281 articles for initial screening (Fig. 2). Upon screening by title and abstract, 1,196 and 37 articles were excluded respectively, resulting in 48 articles meeting the criteria for full-text review. Upon assessment of eligibility, 33 full-text publications were included in this scoping review. In addition, one more record identified through citation searching met the inclusion criteria and was also included.

### Study characteristics

A total of 34 studies were included in this review [23–56]. They were conducted across 10 countries or regions, including the United States (*n* = 19) [25, 28, 29, 33, 34, 37–39, 43, 44, 46–50, 52–55], Australia (*n* = 2) [23, 36], Canada (*n* = 2) [24, 26], Europe (*n* = 2) [40, 42], France (*n* = 2) [27, 32], United Kingdom (*n* = 2) [35, 45], Spain (*n* = 2) [31, 56], Brazil (*n* = 1) [30], Portugal (*n* = 1) [51] and Italy (*n* = 1) [41].

As shown in Table 1, 8 interventional studies involving hospital pharmacists were conducted under a prospective cohort study design (*n* = 6) [23–28], a retrospective study design (*n* = 1) [29] and a case–control intervention study design (*n* = 1) respectively [30]. A total of 1,012 patients undergoing hematopoietic stem cell transplant (HSCT) from 8 hospitals in 5 countries received interventions from hospital pharmacists during treatment. The study sample size ranged between 23 and 460. The duration of intervention ranged from 10 to 299 days.

**Table 1:** Summary of studies involving hospital pharmacist’s interventions in CGT/ATMPs

Author/s Year of publication	Study type	Type of subjects	Study content	Study objectives	Key components of interventions	Major output and outcome	Conclusions or recommendations
Chieng et al. 2013 [23]	Design: A prospective cohort study	Patients undergoing allo-HSCT	*Study location*: Australia *Number of hospitals*: 1 hospital *Number of patients*: 23 (IG 23) *Duration of intervention*: 10 days	To evaluate the effectiveness of a specialty clinical pharmacist working in an ambulatory HSCT clinic	*Pharmacists' interventions*: • *Prescribing*: medication review with a total of six visits • *Administration*: dose administration aids • *Others*: Morisky questionnaire	*Output*: 161 interventions were recorded (high- and medium-risk > 80%, including 32% therapeutic drug monitoring of immunosuppressants and azole antifungals, 15% wrong dose, 12% omitted medication, 12% unnecessary medication, 9% adverse drug reaction *Clinical Outcome*: highly adherent in visit 6 (*p* < 0.0001)	A specialist clinical pharmacist in the SCT outpatient clinic resulted in regular and effective intervention contributing to improved medication management and adherence
Ho et al. 2013 [24]	Design: Non-comparative prospective study	Patients undergoing allo-HSCT	*Study location*: Canada *Number of hospitals*: 1 hospital *Number of patients*: 35 (IG 35) *Duration of intervention*: NA	To determine the effect on medication safety of, as well as potential barriers to, incorporating a pharmacist in the multidisciplinary team of an allo-HCT clinic	*Pharmacists' interventions*: • *Prescribing*: perform medication reconciliation and identify and resolve DRPs, clarify prescriptions and drug coverage issues • *Administration*: medication education to patients and pharmacy consultations to clinic staff, electronic patient record	*Output*: 50 medication discrepancies and 70 DRPs were identified and resolved by pharmacists. Thirty-one of the 70 DTPs resulted directly from a medication discrepancy *Humanistic outcome*: positive satisfaction responses from both patients (average score 4.8/5) and clinic staff (average score 4.6/5)	Pharmacists working as part of the multidisciplinary team identified and resolved medication discrepancies, thereby improving medication safety at the allo-HCT clinic
Alexandar et al. 2016 [25]	Design: Non-comparative prospective study	Patients undergoing bone marrow transplantation	*Study location*: USA *Number of hospitals*: 1 hospital *Number of patients*: 460 (170 inpatients and 290 outpatients) *Duration of intervention*: NA	To assess the impact of clinical pharmacy services in the care of patients undergoing HSCT	*Pharmacists' interventions*: • *Prescribing*: prescription transmission • *Administration*: discharge counseling in the inpatient setting (coordination of insurance, prior authorizations and patient education) • *Others*: an online tool to track the impact of pharmacist services on provider time, four-domain survey tool to assess humanistic outcomes	*Humanistic outcome*: Patients' expectations, experiences, and value perceptions of pharmacists met the predetermined 80% positive response rate *Economic outcome*: an average discharge prescription revenue of $990 per patient through outpatient pharmacy, and 122 h saving through pharmacists' activities	Pharmacists are valuable resources in the care of patients undergoing BMT, as their care translates to increased revenue, provider time savings, and positive perceptions from patients and providers
Defor et al. 2019 [26]	Design: Non-comparative prospective study	Patients with pediatric hematology, oncology, blood and marrow transplant	*Study location*: Canada *Number of hospitals*: 1 pediatric hospital *Number of patients*: 272 *Duration of intervention*: 4 months	To describe key activities performed by a newly deployed clinical pharmacist in an outpatient pediatric hematology, oncology, transplant clinic	*Pharmacists' interventions*: • *Prescribing*: obtain and review the best possible medication history (BPMH), identifying/resolving drug-related problems, medication reconciliation • *Administration*: documentation in patient permanent record, medication counseling, chemotherapy patient (Family) education, medication diary teaching, creating adherence aids, responding drug information questions from staff and families • *Monitoring*: therapeutic drug monitoring, assessment of adherence	*Output*: 1021 interventions were recorded which the most frequent interventions included BPMH, documentation in the patient healthcare record, and counseling patients/families	The integration of a pharmacist into an outpatient pediatric hematology, oncology, transplant clinic resulted in the provision of several key clinical pharmacy services
Charra et al. 2021 [27]	Design 1: Prospective interventional design Design 2: Review (Database: PubMed; Included 6 articles) *	Patients with allo-HSCT	*Study location*: France *Number of hospitals*: 1 hospital *Number of patients*: 61 (IG 26, CG 35) *Duration of intervention*: 100 days	To evaluate the impact of implementing a specialized clinical pharmacy program in patients with allo-HSCT on their adherence to the immunosuppression treatment after discharge	*Pharmacists' interventions*: • *Prescribing*: proactive medication reconciliation, pharmaco-therapeutic analysis of prescriptions, review of medication with patients, identification of DRPs • *Administration*: pharmaceutical consultations, personalized medication intakes schedule, patient education, contact with community pharmacy	*Clinical outcome*: intra-individual variation in immunosuppressant drug serum level higher in IG (*p* = 0.005), but no significant intergroup difference in serum levels, readmissions, acute GvHD or infection (all *P* > 0.05)	The implementation of a specialized clinical pharmacy program for patients who have received allo-HSCT seems to be beneficial for immunosuppression drug adherence
Gawedzki et al. 2021 [28]	Design: Pre-post intervention study	Patients with HSCT	*Study location*: USA *Number of hospitals*: 1 hospital *Number of patients*: 60 (Pre-IG 30, Post-IG 30) *Duration of intervention*: 100 days	To evaluate the clinical impact of a pharmacist driven immunosuppression drug monitoring protocol for HSCT recipients on tacrolimus	*Pharmacists' interventions*: • *Monitoring*: immunosuppression therapeutic drug monitoring which outlined recommended dosing modifications in response to trough levels, organ function, drug interactions and toxicities • *Others*: Standardized dosing protocol	*Clinical outcome*: significant reduction in the number of adverse events (*P* = 0.03*) and increased in the drug interactions (*P* < 0.0001*) and empiric dose adjustment made (*P* = 0.002*), but no significant difference in the percentage of therapeutic tacrolimus levels (*P* = 0.34), nephrotoxicity (*P* = 0.18)	Pharmacist involvement improved safety outcomes such as management of drug interactions and incidence of adverse events in HSCT
Andrick et al. 2022 [29]	Design: Retrospective analysis Study tool: RE-AIM framework	Patients undergoing allo-HSCT	*Study location*: USA *Number of hospitals*: 1 hospital *Number of patients*: 40 (IG 40) *Duration of intervention*: average 299 days	To evaluate the institutional experience building the HCT medication therapy and disease management (MTDM) program	*Pharmacists' interventions*: • *Prescribing*: medication reconciliation at transitions of care • *Preparation*: assist in medication acquisition, Novel Geisinger delivery platform • *Administration*: medication and transplant education, medication therapy management, supportive care management, immunosuppression management, electronic order set preparation, drug information support • *Monitoring*: post-transplantation vaccine compliance, graft-versus-host disease surveillance, infection surveillance, outpatient follow-up • *Others*: Collaborative practice agreement with physician providers, pharmaceutical care satisfaction questionnaire	*Output*: 388 medications were managed in IG: post-transplantation vaccine management (~ 11%), cyclosporine (~ 7%), ursodiol (~ 6%); 2156 DRPs were identified in IG: safety (70%), effectiveness (16%), indication (8%), adherence (4%), education (1%) and cost/insurance (1%); 2959 interventions were conducted in IG: monitoring (29.9%), graft-versus-host disease surveillance (14.8%), dose decrease (5.2%), clarification (4.8%), education (4.3%), immunosuppression management (3.7%); Immunosuppression management: distribution of cyclosporine levels (therapeutic range of 74%) *Humanistic outcome*: Patient satisfaction (71%)	The implementation of an HCT pharmacist service can positively impact patient care
Zanetti et al. 2023 [30]	Design: Case -control interventional study	Patients receiving allo-HSCT	*Study location*: Brazil *Number of hospitals*: 1 hospital *Number of patients*: 61 (IG 33, CG 28) *Duration of intervention*: the period of hospitalization until 100 days after the date of stem cell infusion	To assess if the insertion of the clinical pharmacist in the allo-HSCT team modify the clinical outcomes	*Pharmacists’ interventions*: • *Prescribing*: medication reconciliation at hospital admission, daily revision of the prescriptions, participation in clinical team meetings • *Administration*: production of educational materials on the use of drugs for patients and for the health team, weekly consultations with the patients • *Monitoring*: assessment of adherence, pharmacotherapy monitoring form • *Others*: MedTake instrument to assess knowledge	*Output*: 250 DRPs identified (safety 51.6% and effectiveness 38.4%) and 309 interventions conducted (dose reduction 19.09%, adjustments in administration time 18.12%, education 15.21% and drug removal 10.68%) in IG *Clinical outcome*: no evidence of differences in the transplantation-related mortality, grafting failure, number of readmissions, GVHD, time for grafting and hospitalization time (all *P* > 0.05). Patient adherence improvement (*p* = 0.0115*) *Humanistic outcome*: Patient knowledge improvement (*p* = 0.0001*)	The pharmacotherapy follow-up allowed detecting several DRPs and performing interventions of high clinical relevance and acceptability, in addition to improving adherence and individualizing the pharmacotherapy

*Abbreviations*: *BMT* Bone marrow transplantation, *BPMW* Best possible medication history, *CG* Control group, *DRPs* Drug-related problems, *DTP* Drug therapy problems, *GvHD* Graft-versus-host disease, *HSCT* Hematopoietic stem cell transplant, *IG* Interventional group, *MTDM* Medication therapy and disease management

As shown in Table 2, 26 included studies comprising 7 forms of articles reported quantitative data and qualitative findings, including the review studies (*n* = 11) [32, 34, 35, 37, 46–48, 50, 51, 53, 56], survey studies (*n* = 5, with a total of 320 responses) [38, 41, 42, 45, 54], commentary (*n* = 4) [33, 44, 49, 55], position statement (*n* = 2) [40, 43], report (*n* = 2) [36, 52], communication (*n* = 1) [39], and letter to editor (*n* = 1) [31]. In addition, pharmacist’s roles or related recommended interventions were identified when patients received ATMPs (*n* = 2) [31, 32], HSCT (n = 9) [33–41], HSCT and cellular therapy (*n* = 2) [42, 43], cellular-based therapy (*n* = 1) [44], gene therapy (*n* = 6) [45–50], and CAR T-cell therapy (*n* = 6) [51–56] in the hospital setting. Seven of the included articles encompassed opinions or statements by 3 international professional organizations: the American Society for Transplantation and Cellular Therapy (ASBMT) Pharmacy Special Interest Group (SIG) [33, 37–39, 54], the European Society for Blood and Marrow Transplantation (EBMT) Pharmacist Committee [40] and the American Society for Transplantation and Cellular Therapy (ASTCT) Pharmacy SIG [43].

**Table 2:** Summary of studies involving pharmacists’ roles and responsibilities in CGT/ATMPs

**Author/s Year of publication**	**Type of therapies**	**Study content**	**Study objectives**	**Major insights reported**	**Key roles or interventions mentioned** *Areas of the Medicine Use Process in Hospitals According to the Basel Statement* (A = Procurement, B = Prescribing, C = Preparation, D = Administration, E = Monitoring, F = Training, G = Others)							**Anticipated impact**
					**A**	**B**	**C**	**D**	**E**	**F**	**G**	
Segura et al. 2014 [31]	ATMPs	Design: Letter to editor Location: Spain	To propose advancing hospital pharmacy practice through new competences in ATMPs	Advanced therapies allow enhancing the role of pharmacists in health-system decisions concerning selection, use, and management of medications in hospitals						✓		• Pharmacists' knowledge ( +) in theoretical and practical skills in molecular and cell biology as well as their application to ATMPs
Mebarki et al. 2022 [32]	ATMPs	Design: Review Location: France	To summarize the regulatory framework, hospital and pharmaceutical circuits of ATMPs in Europe and France	The specificity and complexity of ATMPs required a complete reorganization of hospital and pharmaceutical circuits, from patient eligibility to drug administration		✓	✓			✓	✓	• Multidisciplinary collaboration ( +) • Quality and safety of ATMPs use ( +)
Komanduri. 2013 [33]	HSCT	Design: Commentary Location: USA	To illustrate the advances and opportunities to use Collaborative Practice Agreements to improve care	CPAs facilitate the ongoing expansion of clinical services and volume by HSCT centers while maintaining and even increasing the quality of care and patient satisfaction		✓		✓	✓	✓	✓	• CPA implementation ( +)
Merten et al. 2013 [34]	HSCT	Design: Review Location: USA	To provide a framework for implementation of a CPA and address how it may improve HSCT program capacity	The framework for implementation of a CPA consists of conducting a needs assessment, evaluating staffing constraints, identifying training requirements, developing and implementing the CPA				✓			✓	• Multidisciplinary collaboration ( +) • Pharmaceutical care ( +) for HSCT • Patient outcomes assessment ( +) • Quality of patient care ( +)
Bauters et al. 2014 [35]	Paediatric haemato-oncology SCT	Design: Review Location: UK	To summarise the sparse literature data on clinical pharmacy activities in the paediatric HSCT setting augmented with advice from expert opinion	HSCT pharmacists are well-positioned to take a lead role in patient assessment and the development and implementation of guidelines for supportive care				✓	✓	✓		• Pharmaceutical care ( +)
Cheing et al. 2014 [36]	allo-HSCT	Design: Report Location: Australia	To describe the implementation of a clinical pharmacy service to ambulatory HSCT patients	The clinical pharmacist's presence in the ambulatory setting resulted in the identification and rectification of many medium to high risk medication related problems and improved overall patients' adherence		✓		✓	✓			• Ambulatory clinical pharmacy services ( +) • Patient outcome assessment ( +) • Quality of patient care ( +)
Clemmons et al. 2018 [37]	HCT	Design: Review Location: USA	To clarify the various services pharmacists can provide in the multidisciplinary care of HCT patients and describe the various potential roles and responsibilities of the HCT pharmacist	Pharmacists who are trained in hematology/oncology and HCT can provide a positive impact through providing direct management of the complex medication regimens, contributing to multidisciplinary education, and engaging in research efforts		✓		✓	✓	✓	✓	• Quality of patient care ( +) with economic value, humanistic value and patient clinical outcomes
Bryk et al. 2019 [38]	HSCT	Design: Survey study Location: USA Tool: An electronic questionnaire developed by the Advocacy & Policy Working committee of the ASBMT Pharmacy SIG Number of *responses*: 48 from 40 different institutions	To assess the current state of CPAs already in place between oncologists and clinical pharmacists in the HCT setting	Collaborative practice agreements allow HCT pharmacists to more independently and efficiently contribute to the work of the multidisciplinary team caring for patients undergoing HCT		✓		✓	✓	✓	✓	• CPA implementation ( +) including pharmacist recognition as providers on a national level and consistent mechanisms for reimbursement for services provided
Clemmons et al. 2020 [39]	HSCT	Design: Communication Location: USA	To stimulate further research to justify the roles of HCT pharmacists and the correlation of such research to various outcome measures	The existing data support the vital role of HCT pharmacist in various quality metric, clinical, humanistic, and financial outcomes		✓		✓	✓	✓		• Pharmacy practice ( +) with report and assessment • Patient outcome assessment ( +)
Langebrake et al. 2020 [40]	HSCT	Design: Position statement Location: EU Organization involved: the European Society for Blood and Marrow Transplantation (EBMT) Pharmacist Committee	To give advice for the roles and responsibilities of transplant clinical pharmacists/pharmacologists (CP/Ps) and to advocate for this role within European transplant centers	This paper reflects the recommendations by the members of the EBMT Pharmacist Working Committee and highlights the roles and competencies of clinical pharmacists and pharmacologists involved in HSCT		✓	✓	✓	✓	✓	✓	• Pharmacists' knowledge ( +) • Pharmacy practice ( +) • Quality of patient care ( +)
Faraci et al. 2021 [41]	HSCT	Design: Survey study Location: Italy *Tool*: A 63-item online questionnaire *Number of responses*: 52 from Italian HSCT centres	To evaluate the involvement of pharmacists in the HSCT program in Italian adult and paediatric centres	The survey highlighted very good collaboration between pharmacists and haematologists (80.8%) while less frequent collaboration with nurses (50%)		✓	✓	✓				• Quality of patient care ( +)
Duncan et al. 2023 [42]	HSCT and cellular therapy	*Design*: Survey study Location: EU *Tool*: A 47-item online questionnaire based on consensus recommendations designed by EBMT Pharmacist Committee *Number of responses*: 84 from 27 member countries (highest responses countries: UK, Belgium and Spain)	To assess the current standards of clinical pharmacist/pharmacologist services across the EBMT in relation to transplant activity, staffing, and degree of involvement in the roles described in this key guidance document	The ever-expanding role of pharmacists and pharmacologists in the management of transplant and cellular therapy recipients		✓	✓	✓	✓	✓		• Pharmacy services ( +)
Mahmoudjafari et al. 2020 [43]	HCT and Cellular Therapy	*Design*: Position statement *Location*: USA *Organization involved*: American Society for Transplantation and Cellular Therapy Pharmacy Special Interest Group	To provide pharmacy practice management and clinical management recommendations for COVID-19 in HCT and cellular therapy recipients	The statement emphasized the importance of pharmacists' role in the health care team which includes continue to monitor patients, provide clinical recommendations, and provide critical education to patients in need		✓		✓	✓			• Pharmacy clinical services ( +) for patient receiving HCT and cellular therapies during the COVID-19 pandemic
Blind et al. 2023 [44]	Cellular-based therapies	*Design*: Commentary *Location*: USA	To call for pharmacies to take a multidisciplinary approach in standardizing the therapeutic handling and administration of all CBTs within the institution	The health-system pharmacist will have an integral part of reinforcing patient rights of medication administration, especially in Biologic drug management, Multidisciplinary team coordination and Supportive care management	✓	✓	✓	✓	✓	✓		• Education resources ( +): • Infrastructure resources ( +) • Operational plans developed ( +)
Stoner et al. 2018 [45]	Gene therapy medicinal products	*Design*: Editorial including a survey *Location*: UK *Tool*: An online questionnaire based on the European Guidance for hospital pharmacists on handling licensed GTMPs *Number of responses*: 84 from 73 hospitals (79 pharmacists and 3 pharmacy technicians)	To review of pharmacy facilities and implementation of ATMP in the UK	Most hospital pharmacy departments do not have aseptic facilities for the reconstitution of GTMP, or have the appropriate freezers in place. Staff do not have the understanding or training of these products unless they are experienced in using them in clinical trials	✓		✓	✓		✓		• Multidisciplinary collaboration ( +) • Pharmacy practice ( +)
Blind et al. 2019 [46]	Viral-mediated gene therapy and genetically modified therapeutics	*Design*: Review *Location*: USA	To provide recommendations for occupational safe drug handling applicable to the manipulation of viral vectors in a health-system pharmacy	The 2 guidance documents provide the necessary biosafety handling principles in the manipulation of biological material			✓		✓	✓		• Pharmacy practice ( +) • Healthcare providers and environment safety ( +)
Petrich et al. 2020 [47]	Gene replacement therapy	*Design*: Review *Location*: USA	To comprehensive review of gene replacement therapy with guidance and expert opinion on handling and administration for pharmacists	Pharmacists have a key role in the proper handling and general management of gene replacement therapies, identifying risk level, establishing infrastructure, and developing adequate policies and protocols			✓	✓		✓		• Healthcare providers and environment safety ( +) • Pharmacists’ knowledge and skills ( +)
Canfield et al. 2021 [48]	Gene therapy	*Design*: Review *Location*: USA	To provide health systems with considerations that health-system pharmacies and specialty pharmacy programs may reference when evaluating and implementing services around gene therapies	All treatment centers provided VN Ocular gene therapy care are within academic centers, integrated delivery networks or health systems. Health-system pharmacy departments is continued to be a prominent care provider for patients receiving gene therapy	✓	✓	✓	✓		✓	✓	• Pharmacists’ knowledge and skills of gene therapies ( +)
Myers et al. 2021 [49]	Gene therapy	*Design*: Commentary *Location*: USA	To provide a background on gene therapy and identify a critical gap in pharmacy practice education	There are few gene therapy training resources for pharmacists, and gene therapies require complex handing and administration						✓		• Pharmacists’ knowledge of gene therapies ( +) • Pharmacy practice standards ( +)
Hernandez et al. 2022 [50]	Viral Vector Gene Therapy	*Design*: Review *Location*: USA	To overview the multifaceted biosafety points that pharmacists and pharmacies must consider when providing viral vectors gene therapy	Pharmacists must be educated and ready to comply with biosafety standards in order to provide these treatments for their patients			✓	✓		✓	✓	• Healthcare providers’ knowledge of viral vector gene therapy ( +) • Participation in the development of facility policies and biosafety handling guidelines ( +)
Braga et al. 2021 [51]	Immunocellular therapy with CAR T-cell	Design: Review *Location*: Portugal	To review the key aspects of CAR T-cell medicines and to describe the hospital pharmacist’s role within the multidisciplinary health team	Hospital pharmacists have a responsibility to contribute to CAR T-cell medicines rational use, as well as being an active member of the multidisciplinary clinical team to manage and follow-up patients	✓		✓	✓	✓	✓		• Multidisciplinary collaboration ( +)
Dangi-Garimella et al. 2017 [52]	CAR T-cell therapy	*Design*: Report through conversation with a pharmacist *Location*: USA	To share pharmacist's experiences with CAR T-cell therapy in the clinic	Pharmacist's change may be needed for a safe, effective adoption of the new treatment modality in the oncology clinic		✓		✓	✓	✓		• Pharmacy practice in managing CRS ( +) • Pharmaceutical care ( +)
Dushenkov et al. 2019 [53]	CAR T-cell therapy	*Design*: Review *Location*: USA	To describe Risk Evaluation and Mitigation Strategy (REMS) requirements for Kymriah™ and Yescarta™ in relation to practice of pharmacy	As active members of multidisciplinary clinical teams, pharmacists are likely to be responsible for the execution of CAR T-cell therapies' REMS programs		✓	✓	✓	✓	✓		• Pharmacists’ knowledge of CAR T-cell therapy ( +)
Mahmoudjafari et al. 2019 [54]	CAR T-cell therapy	*Design*: Survey study *Location*: USA *Tool*: An online questionnaire from consensus recommendations designed by ASBMT Pharmacy SIG *Number of responses*: 52	To gain insight into the infrastructure and practices on the current administrative, logistic, and toxicity management practices of CAR T cell therapy across the United States	Absorbing the energy of CAT T cell therapy has challenged HSCT programs across the country to strengthen department infrastructure, develop new committees and policies, and implement significant education to ensure safe administration		✓	✓		✓		✓	• Pharmacy practice ( +) with the current administrative, logistic, and toxicity management guidelines
Booth et al. 2020 [55]	CAR T-cell therapy	*Design*: Commentary *Location*: USA	To summazise the pharmacist’s role in chimeric antigen receptor T cell therapy	Pharmacy involvement in the implementation and maintenance of CAR T cell therapy program emphasizes the importance of pharmacy involvement as part of a multidisciplinary care	✓		✓	✓	✓	✓	✓	• Pharmacists’ knowledge and skills of CAR T-cell therapies ( +)
Moreno-Martínez et al. 2020 [56]	CAR T-cell therapy	*Design*: Review *Location*: Spain	To describe the oncohematological pharmacist’s role within the multidisciplinary clinical team	CAR-T therapy offers the hospital pharmacist the opportunity to work closely with the rest of the clinical professionals involved in the process, allowing their contribution to the development of procedures, clinical practice guidelines of global approach	✓	✓	✓	✓	✓	✓	✓	• Multidisciplinary collaboration ( +) • Quality of patient care ( +) • Operational procedure standards ( +)

*Abbreviations*: *ASBMT* American Society for Transplantation and Cellular Therapy, *CP/Ps* Clinical pharmacists/pharmacologists, *CPAs* Collaborative Practice Agreements, *EBMT* European Society for Blood and Marrow Transplantation, *HCT* Hematopoietic cell transplantation, *HSCT* Hematopoietic stem cell transplant, *REMS* Risk Evaluation and Mitigation Strategy, *SIGs* Special interest groups

^*^ ( +) indicates a positive effect on expected outcomes if managed by hospital pharmacists

### Hospital pharmacists’ interventions for patients receiving CGT/ATMPs

Of the eight studies included in the Table 1, six were conducted in hospital settings [24, 25, 27–30], one study took place in an ambulatory HSCT clinic [23], and another one was conducted in a pediatric hospital [26]. All subjects involved were patients undergoing HSCT. The most common interventions for patients undergoing HSCT provided by hospital pharmacists focused on medicine administration (*n* = 8) [23–30], prescribing (*n* = 7) [23–27, 29, 30], and monitoring of medicines use (*n* = 4) [26, 28–30]. Education and counseling were the most common pharmacist services related to the medicine administration in these 8 studies. In addition to providing medication and transplant education [24–27, 29, 30], medication counseling at admission and discharge to patients [25–27, 29, 30], pharmacists actively provided pharmacy education and counseling to other healthcare providers [24, 26, 27, 30].

The process of medication therapy management by pharmacists covered various implementation details, including creating dose administration aids [23], adherence aids [26], personalized medication intakes schedule [27], and standardized dosing protocol [28]. Apart from administration, they performed medication reconciliation [24, 26, 27, 29, 30], identified and resolved drug-related problems (DRPs) [24, 26, 27] to optimize each prescribed medication use. In addition, pharmacists reviewed and validated the medications according to the HSCT patients’ complex combination use of medications [23, 26, 27, 30].

As a member of the clinical team, pharmacist collaborated with other healthcare providers to adjust the prescriptions [30]. Pharmacists also participated in monitoring of immunosuppression therapeutic drug which outlined recommended dosing modification to trough levels, organ function, drug interactions and toxicities [28]. Andrick et al. reported the pharmacist interventions of monitoring the post-transplantation vaccine compliance, graft-versus-host disease and infection surveillance, outpatient follow-up to improve medication safety [29].

Among the 8 studies described in the Table 1, a total of 4,141 interventions and 2,226 DRPs were recorded, identified and resolved by hospital pharmacists. Interventions mentioned in the included studied are listed in order of frequency: therapeutic drug monitoring [23, 29, 30], dose reduction [23, 29, 30], patient education [26, 29, 30], immunosuppression management [23, 29], drug removal [23, 30], medication history reviewing [26] and graft-versus-host disease surveillance [29]. Some studies reported the identified DRPs during interventions, which mainly involved safety and effectiveness issues [29, 30].

Three studies reported clinical outcomes, with one study showing a high level of adherence in patient visits (*p* < 0.0001) [23], another study demonstrating a significant reduction in the number of adverse events (*P* = 0.03) and an increase in the empiric dose adjustment made (*P* = 0.002) [28], and another study revealing an increase in immunosuppressant drug serum levels with intra-individual variation (*p* = 0.005) [27]. Patient strong satisfaction responses were considered as a primary humanistic outcome [24, 25, 29]. Zanetti et al. [30] also reported a significant improvement in patients’ knowledge and adherence (*p* < 0.05). Additionally, Alexander et al. [25] reported the positive economic outcomes of pharmacists in the care of bone and marrow transplant patients, including increased discharge prescription revenue and savings in pharmacist activities hours. Further details are provided in Table 1.

### Key roles of hospital pharmacist in CGT/ATMPs

As shown in Table 2, the key roles of hospital pharmacists in supporting appropriate and safe use of CGT/ATMPs identified from the 26 included studies were summarized. These encompasses all six duty domains indicated in the Basel Statement. Perspectives and findings regarding the hospital pharmacists’ role in CGT/ATMPs management identified from the literature encompassed key components of interventions, covering procurement (*n* = 6) [44, 45, 48, 51, 55, 56], influences on prescribing (*n* = 16) [32, 33, 36–44, 48, 52–54, 56], preparation and delivery (*n* = 15) [32, 40–42, 44–48, 50, 51, 53–56], administration (*n* = 21) [33–45, 47, 48, 50–53, 55, 56], monitoring of medicines use (*n* = 17) [33, 35–40, 42–44, 46, 51–56], human resources, training and development (*n* = 21) [31–33, 35, 37–40, 42, 44–53, 55, 56], and other interventions beyond the Basel Statement (*n* = 11) [32–34, 37–40, 48, 54–56]. More details about the role of hospital pharmacists in managing the different types of therapies or medical products can be seen in the Table 3.

**Table 3:** Components of hospital pharmacists’ interventions reported in CGT/ATMP

Components of intervention reported	Description	Detailed examples in different therapies				
		**ATMP** (*n* = 2)	**HSCT** (*n* = 9) **& HSCT and cellular therapy** (*n* = 2)	**Cell therapy** (*n* = 1)	**Gene therapy** (*n* = 6)	**CAR T-cell therapy** (*n* = 6)
**Theme 1 Procurement** (*n* = 6)	Ordering management (*n* = 4) [44, 48, 51, 56]			• Ordering management [44]	• Financial and purchasing systems [48]	• Acquisition of medicines containing CAR T-cells [51] • Participation in the selection and approval of the CAR T medication [56]
	Procurement oversight (*n* = 3) [45, 48, 55]				• Oversight GTMPs’ procurement [45] • Sign-off procedures at an executive level [48] • Assessment of the implications of internal/external product acquisition methods [48]	• Procurement management [55]
**Theme 2 Influences on Prescribing** (*n* = **1**6)	Medical review and validation (*n* = 13) [32, 33, 36–40, 42, 44, 52–54, 56]		• Medication history review [32, 33, 36–39, 42] (eg. check pathology test results and pharmacy dispensing records [36]) • Assess the appropriateness of the current medication [40] • Manage chemotherapy processes and anti-infective therapies [33, 37–39] • Pretransplant workup and verification of conditioning regimens [40, 42]	• COPE verification and concomitant therapy review [44] • Pre-conditioning assessment [44]		• Develop order sets [52]; • Pharmacy Order Entry System usage [53] • Formulary management [52, 54] (eg. add toxicity treatment medications, including siltuximab and tocilizumab, product approval for formulary addition) • Clinical data review and validate for treatment plan [54, 56]
	Drug related problems (DRPs) identified and resolved (*n* = 6) [33, 37–39, 41, 42]		• Evaluate or report transplant-related outcomes [33, 37–39] • Identify and solve DRPs [33, 40–42] (eg. untreated indication, subtherapeutic or supratherapeutic dosages);			
	Multidisciplinary collaboration to improve prescribing (*n* = 8) [32, 33, 40–43, 48, 56]	• Communication and coordination with numerous actors [33]	• Analysis of DRPs with HCPs [33, 41] • Collaboration with HCPs in formularies' decision-making [40, 42, 43]	• Coordination of medication use process for patient dosing [45]	• Engaged with internal physician expertise [48]	• Support treatments [56]
	Medical reconciliation (*n* = 4) [36, 42, 43, 52]		• Medical reconciliation to monitor for changes in regimen [36] • Medication reconciliation at admission and discharge [42] • DDIs evaluated and medication reconciliation remotely [43]		• Medication reconciliation with various care providers [52]	
**Theme 3 Preparation and Delivery** (*n* = 15)	Receipt, control, storage, reconstitution, and distribution (*n* = 12) [32, 40–42, 44–46, 51, 53–56]	• The receipt, control, storage and reconstitution before final dispensation [32]	• Preparation of cytotoxic, document procedures for the use of ATMP [40]; product availability review [41, 42]	• Inventory management [44] • Product handling, storage, preparation and delivery [44]	• GTMPs handling and delivery [45] • Quality ensurance of GTMPs for the intended use [45] • Evaluation of the need for storage of serum samples and vaccines [46] • Engineering control of sterile hazardous drug compounding [46]	• Confirmation of the availability of CAR T-cells and receipt of medicines [51, 56] • Monitoring storage and transport conditions [51, 53–56] • Check the integrity of the medicine/labeling/certificate of analysis (CoA) [52] • Double dispensing verification [51] • Pharmacy labeling and handling [55, 56]
	Preparation for the dedicated premises and equipment (*n* = 8) [32, 45–48, 50, 51, 56]	• Preparation for the dedicated premises and equipment [32]			• Environmental control [45, 47, 48, 51] (eg. biosafety cabinet hoods cleaning before and after viral vector doses); storage access limited to the trained staff [46] • Equipped with appropriate freezers and gene therapy aseptic facilities [45, 50] (eg. facilities met the BSL-1 and BSL-2 requirements), personal protective equipment [46]	• Defrosting SOP [56]
**Theme 4 Administration** (*n* = 21)	Medication education to patients and caregivers (*n* = 11) [33, 35–40, 42, 47, 50, 55]		• Patients/caregivers education [33, 35–40, 42] (eg. administration instructions, missed dose instructions, side effect management, serious complications)		• Education about administration issues, waste handling and any other issues specific to the particular gene therapy [47] • Education on risk factors (viral shedding) [50]	• Patient education [55]
	Medication counseling to patients and caregivers (*n* = 9) [33, 35–40, 42, 43]		• Medical counseling to patients and caregivers [33, 35–40] (eg. dosing schedules, administration. instructions, and side effects) • Admission and discharge medication [35, 36, 43] • Drug information service [40, 42]			
	Pharmacy education or counseling to other HCPs (*n* = 8) [33, 35–39, 42, 44]		• Healthcare providers education [34, 36–40, 43] (eg. HCT medications, management of toxicities, and transplant-related complications, conditioning regimens and management of toxicities from immunosuppressant medications)	• Healthcare providers education [45]		
	Medication therapy management (MTM) (*n* = 12) [33–35, 37–42, 52, 55, 56]		• MTM assist with transitions of care [34, 38–40] • MTM visits [35] • Implementation and maintaining high-quality patient care programme [36] • Prospective medication management [41, 43] • Chemotherapy drugs and parenteral nutrition management [42] • Consideration of patient preferences [43]		• Management of the logistics of the pharmacotherapy and toxicities of CAR T cells [53]	• Medication administration and supportive care [56] • MTM quality tracking [56] • Confirmation with the preprocessing phase [57]
	Documentation order (*n* = 7) [40, 42, 43, 48, 50, 53, 55]		• Documentation DRPs and pharmacists’ interventions [41] • Computerized physician order entry with clinical decision support system [41, 43, 44] • EMR and supporting technology build [44]		• Standardized product nomenclature within clinical and financial documentation systems [49] • Drug entry built into the electronic medical record system [51]	• Computerized order entry incorporated with clinical decision support rules [53, 56]
	Medical history assessment (*n* = 4) [40, 42, 51, 56]		• Assessment of medical history and information [41, 43]			• Analysis of the patients' clinical information in the selection process [52, 57]
	Administration quality and process improvement (*n* = 8) [36, 40, 43, 47, 48, 50–52]		• Evaluation of medication adherence using a dosette box and Morisky questionnaire [37] • Quality and process improvement [41] • A rotating model of working remotely for pharmacists [44]		• Formation of clinical biosafety committees [48] • Standardized product nomenclature within clinical and financial documentation systems [49] • Staff exposure logs to track all parties that work with genome product [51]	• Additional control systems [52] • Management of the logistics of the pharmacotherapy and toxicities of CAR T cells [53]
**Theme 5 Monitoring of Medicines Use** (*n* = **1**7)	Therapeutic drug monitoring (TDM) (*n* = 12) [33, 35–40, 42–44, 46, 51]		• Assist with TDM and symptom management [34, 38–41, 43, 44] • Optimize GvHD management and facilitate post-transplant vaccination [34, 38–40] • TDM of immune suppressive or antibiotics [36] • Adherence to prescribed medication regimen [37] • Continued monitoring of high-risk complex patients [44] • Pharmacokinetic monitoring and adjustment of renal and hepatic [44]	• Assist with TDM and dose adjustments [45] • Infection prophylaxis [45]	• Medical surveillance to any individual that regularly handles the agents [47]	• A centralized EU patient registry to monitor long-term safety and effectiveness [52] • Local clinical guidelines to refer the PV and monitoring [52]
	AE reactions (*n* = 14) [33, 35–40, 44, 51–56]		• Monitor transplant-related outcomes [34, 37–40] • Etection of adverse reactions or problems with administration of intravenous drugs [36, 37] • Identify/report/record/prevent of AEs and medication errors [41]	• AE management [45]		• AEs monitoring and record (focus on the cytokine release syndrome (CRS)) [52, 56, 57] • Management of the CRS and CRES (CAR T-cell-related encephalopathy syndrome) [53, 57] • Monitoring with dose adjustment [54] • Toxicity prophylaxis and documentation (seizure/infection/growth factor) [55]
	Pharmacist rounds (*n* = 7) [33, 35, 37–40, 42]		• Participation in interdisciplinary rounds [34, 36, 38–41, 43]			
	Follow up (*n* = 3) [43, 51, 56]		• Telephone follow up [44]			• Well-defined follow-up plan [52] • Patient follow-up after discharge [57]
**Theme 6 Human Resources, Training and Development** (*n* = **21**)	Staff training (*n* = 16) [31–33, 35, 37–40, 42, 45, 46, 50–53, 56]	• Training program to advance in proficient competences with ATMPs management, implementation, and research [32] (eg. vehicle delivery, pharmaceutical compounding, quality control, regulatory framework, pharmacovigilance, biosafety or risk assessment) [32, 33]	• Institutional and collaborative research and scholarly activities [34, 38–40] • Student education [41, 43] • Adequate education and training in paediatrics and cancer [36]		• GTMP handling training [46] • Biosafety Handling training and shipping training [47] • Gene therapy training [50] • Training the appropriate precautions [51]	• Training in the treatment of cellular therapies [52, 53] • Training in location, preparation, and timely dispensing of tocilizumab [54] • Special safe handling procedure education [57]
	Staff recruitment (*n* = 1) [32]	• experienced pharmacists recruitment [33]				
	Related guidelines or SOP developed (*n* = 13) [33, 35, 37–40, 42, 44, 47, 50, 53, 55, 56]		• Assist with development and implementation of guidelines and SOPs (eg. acute effects or toxicities and late effects or toxicities [36], HSCT and supportive care [34, 38–41, 43], patient assistance program [40])	• Operational plans: strategized path for delivery of therapies (formulary intake process and financial considerations), policies and processes for biohazardous product handling and preparation, SOP procedures. [45]	• Development of the consensus guidelines and procedures [48] • Facility policies to address all aspects of dosing [51]	• Standarized protocols/guidance/ policy development [54, 56, 57] (eg. guide on indications and therapy criteria with CAR T-cells) • Preparation and administration protocol (drug stored in refrigerated automated dispensing cabinets) [54]
	Core team building (*n* = 1) [48]				• Development of a core team to lead service implementation with key stakeholders [49]	
**Others (*n* = 11)**	/	• Authorized agreement of the Health Regional Agency for CAR T cells activity [33]	• Collaborative practice agreements (CPA) [34, 35, 39, 40] • HCT Clinical Pharmacist Role Description statement [34, 38–40] • Tthe role and competencies of the EBMT CP/P [41] • Pharmacoeconomics [41]		• Pharmacy leadership involved in health system-level payer relations discussions relevant to gene therapy [49]	• Practice of product reimbursement and process of financial investigation [55–57] • Authorized Representative with REMS program [56]

*Abbreviations*: *AE* Adverse events, *BSL* Biosafety Level, *CoA* certificate of analysis, *CRES* CAR T-cell-related encephalopathy syndrome, *CPA* Collaborative Practice Agreements, *CP/Ps* Clinical pharmacists/pharmacologists, *CRS* cytokine release syndrome, *DDIs* Drug-drug interactions, *DRPs* Drug related problems, *HCP* Healthcare professional, *MTM* Medication therapy management, *PV* Pharmacovigilance, *SOP* Standard operating procedure, *TDM* Therapeutic drug monitoring

As shown in Table 3, 6 studies highlighted the role of pharmacists in the procurement management and oversight of cell and gene therapy products [44, 45, 48, 51, 55, 56], including participation in the selection and approval of the CAR T medication [56], as well as the assessment of the acquisition systems or methods [48]. Due to the complexity of these advanced therapies and medical products in the practical use, 13 studies reported the role of pharmacists in medical review and validation [32, 33, 36–40, 42, 44, 52–54, 56], such as medication history review and concomitant therapy review. For patients undergoing HSCT, hospital pharmacists participated in the pretransplant workup and verification of conditional regimens [40, 42] and the management of chemotherapy processes and anti-infective therapies [33, 37–39], as well as identified and solved drug related problems (DRPs) during the transplant processes [33, 37–39, 41, 42].

In the formulary management of CAR T-cell therapies, pharmacists put more attention to the inclusion of toxicity treatment medications (eg. siltuximab and tocilizumab) [52, 54] and seek product approval for prescription additions [54]. As a member of medical multidisciplinary team, hospital pharmacists were recommended to collaborate with other healthcare providers to improve the quality of prescribing [32, 33, 40–43, 48, 56]. Four studies reported that hospital pharmacists also ensured the medical reconciliation to monitor regimen changes at admission and discharge [36, 42, 52], even only remotely [43].

During the pharmacists’ intervention of the preparation and delivery procedures, receipt, control, storage and reconstitution of the specific biologics or cytotoxic drugs [32, 40–42, 44–46, 51, 53, 55, 56], as well as the preparation for the dedicated premises and equipment [32, 45, 46, 50, 56], were reported in 14 of the included studies. Most of the pharmacy practice related to medicine administration included in this review was through education and counseling to patients, caregivers and other healthcare providers [33, 35–40, 42–44, 47, 50, 55]. This was mainly to provide information about dosing administration and instruction, side effect management, serious complications, admission and discharge medication plan.

Pharmacists also provided various medication therapy management (MTM) [33–35, 37–42, 52, 55, 56] or conducted MTM quality tracking [55] for patients to implement and maintain high-quality pharmaceutical care. Physician order documentation and the utilization of the electronic order entry system by pharmacists were mentioned in the included 7 studies [40, 42, 43, 48, 50, 53, 55]. Additionally, pharmacists devoted to improving the administration quality and processes [36, 40, 43, 47, 48, 50–52], such as through evaluating the medication adherence [36], creating pharmacist rotating model [43], formatting the clinical biosafety committees [47].

Pharmacists recorded the patients’ relevant responses to the therapeutic drug management (TDM) [33, 35–40, 42–44, 46, 51], adverse drug reactions [33, 35–40, 44, 51–56], pharmacy rounds [33, 35, 37–40, 42] and follow-up visits [43, 51, 56] for monitoring purpose. Pharmacist provided a variety of monitoring interventions for HSCT patients to improve the quality of care, including optimizing GvHD management [33, 37–39], facilitating the post-transplant vaccination [33, 37–39], managing immune suppressive or antibiotics [35, 44], monitoring the pharmacokinetic of renal and hepatic [43]. As for using CAR T-cell products, pharmacists paid more attention to monitor and record the cytokine release syndrome (CRS) [51, 55, 56] and CAR T-cell-related encephalopathy syndrome (CRES) [52, 56].

The intervention of pharmacists in the management of CGT/ATMPs are inseparable from the improvement of their own knowledge and skills, most of which focused on staff training [31–33, 35, 37–40, 42, 45, 46, 49–53, 56], staff recruitment [32] and core team building [48]. These training programmes included disease education [35], ATMPs management, implementation and research (eg. vehicle delivery, pharmaceutical compounding, quality control, regulatory framework, pharmacovigilance, biosafety or risk assessment) [31, 51, 52], biosafety handing and shipping training [45, 46, 53, 56], and precautions training [50]. Furthermore, 13 studies [33, 35, 37–40, 42, 44, 47, 50, 53, 55, 56] reported that pharmacists had an important role in assisting with the development and implementation of related guidelines and standard operating procedures (SOP), including patient assistance program [39], HSCT and supportive care guidance [33, 37–40, 42], consensus guidelines and procedures [47], facility polices [50], preparation and administration protocol [53].

As for the interventions beyond the duty domains described in the Basel statement, 5 studies mentioned the roles and competencies of hospital pharmacists from the HCT Clinical Pharmacist Role Description statement developed by ASBMT Pharmacy SIG [33, 37–39] and consensus recommendations involved in HSCT of EBMT clinical pharmacist and clinical pharmacologist (CP/P) [40]. Collaborative practice agreements (CPA) were used to standardize the pharmacists’ intervention in TDM between clinical pharmacists and collaborating physicians [33, 34, 38, 39]. Financial related interventions were also considered, including pharmacoeconomic [40], pharmacy leadership involved in health system-level payer relations discussions [48] and practice of product reimbursement and process of financial investigation [54–56].

## Discussion

This scoping review summarized the critical roles of hospital pharmacists in providing interventions for CGT/ATMPs and identified evidence of positive outcomes for patients undergoing HSCT treatment. Although empirical studies on the role of hospital pharmacists in managing ATMPs are lacking, insights can be drawn from their roles in HSCT. Overall, this study presents a comprehensive blueprint for interventions provided by hospital pharmacists for these innovative pharmaceutical products and complex therapies, underscoring their integral role as key members of the healthcare team. Pharmacists are increasingly involved in the use of innovative advanced therapies, and their role is gaining greater recognition from multiple stakeholders and professional organizations. The value of pharmaceutical care in the field of CGT/ATMPs is gradually emerging, potentially exerting positive impacts on patients’ clinical, economic, and humanistic outcomes. Actions are needed to ensure hospital pharmacists’ competence in accordance with prescribed practice standards, supporting the development and strengthening of their roles.

### The integrated role of hospital pharmacists in the management of CGT/ATMPs

Hospital pharmacists play an integrated role in supporting the appropriate and safe use of the CGT/ATMPs. Interventions by hospital pharmacists for HSCT patients identified in this review mainly involved prescribing, administration and monitoring, with less emphasis on procurement, preparation and training. The recorded HSCT-interventions by pharmacists had been shown to be beneficial to patient care in terms of related healthcare output (e.g. medication discrepancies and DRPs [24, 30], immunosuppression management [27], MTM [29]), as well as clinical outcomes (e.g. medication adherence [23, 30], immunosuppressant drug serum level [27]), humanistic outcomes (e.g. patients’ satisfaction [24, 25, 29] and knowledge [30]) and economic outcomes (prescription cost, pharmacists’ hours saving [25]).

In addition, the role of hospital pharmacists in HSCT, cellular therapy, gene therapy, and the treatment of ATMPs has been recognized by multiple stakeholders and professional organizations. As reported in the included studies in this review, hospital pharmacists have the responsibility of supporting the management and care of patients requiring CGT/ATMPs by providing practices in procurement, prescribing, preparation, administration, monitoring and human resources development. Interventions provided by pharmacist might be categorized as CGT/ATMPs-based (eg. procurement management and oversight, transport and preservation, environment and equipment assessment, handling and delivery), direct to patient-based (eg. education and counseling to patient, DRP and ADR identified and resolved, medical review and reconciliation, TDM monitoring and pharmacy rounds), and HCPs-based (eg. training and collaboration, process improvement).

Additionally, pharmacy departments are recommended to identify and engage with health-system level payer relations or managed care leaders [48]. In the United States, payer coverage for gene therapy may fall under medical benefits, pharmacy benefits, or both and may involve complex authorization processes. Collaborating with key payer decision-makers, particularly before potential FDA actions, may enhance HCP team members’ understanding of the payer processes. Pharmacy leaderships are also encouraged to establish oversight systems or sign-off procedures at the executive level to obtain high-cost pharmaceuticals. Even when opting for products from external specialty pharmacies, considerations must be given to costs associated with product management as well as the risks related to product storage, handling, and oversight [48].

Patients seeking care of such therapies and medical products in hospitals were usually prone to more serious medical conditions (such as cancer or genetic diseases) requiring more complex medication use [15, 18]. Assessing the complete medical and medication history of patients leading to advice about MTM and TDM is considered important for both prescribers and patients [57]. By reviewing and validating the rationale of medication use, pharmacists were empowered to identify and prevent the avoidable AEs and ADRs, and to monitor any unforeseen DRP for effective management. Informing the patients about the risks and common side effects of cell and gene therapy, including GvHD and infection after HSCT [33, 37–39], occurrence of CRS and CRES during the CAR T-cell therapy [51, 52, 55, 56], or related toxicities and immunological risks [35, 43–45, 54], was some of the pharmacists’ interventions when providing care. However, program design involving the role of pharmacists in ATMPs required more research investigating its effect on patient outcome, which would further encourage physicians and patients to improve the evidence for acceptance of pharmacist interventions.

Additionally, pharmacist interventions that focused on collaborative practice and pharmacy practice innovation in broader contexts were also critical to advancing the interprofessional role of hospital pharmacists for it to become more prominent and meaningful [58]. The implementation of CPA promotes the collaborations between pharmacists and physicians, as well as to contribute their unique expertise to patient care and other health-related processes [33, 34, 48, 49]. The enhancement of pharmacist competence would benefit HSCT recipients in accordance with the development and implementation of CPA framework [33, 34]. Practice innovation, which is mainly manifested in the leadership of pharmacists in the exploration, implementation, and standardization process of services, encompasses the expansion and strengthening of current roles, or services and practice models, as well as the development of new roles within the existing practice settings [59].

### The value-based pharmaceutical care of managing CGT/ATMPs

Providing high-quality patient care in an efficient health care system will reduce healthcare costs to provide value [60]. In the development and implementation of hospital pharmacy services, considering the metrics that demonstrate the value of pharmaceutical care is regarded as very important. Value-based metrics are helpful in providing evidence of the rationality of resources to support pharmacists in expanding their service. Pharmacist can show the value-based impact in the economic, humanistic and patient clinical outcomes.

From the perspective of economic value, positive economic outcomes are manifested in revenue generation and cost reduction. One such pharmacy services is coordination of discharged medications, which allows pharmacists to play a role in critical care of transitions for patients receiving HSCT who often bear a higher burden of medications [25]. For example, Alexander et al. reported that pharmacists in the ambulatory care setting at their healthcare institution in the United States were billed for clinical services through facility fees. Pharmacy services provided to HSCT patients resulted in the generation additional revenue through pharmacy billing, time savings for providers, and outpatient pharmacy prescription referrals [25].

In addition, the medication therapy management operated by credentialed pharmacists engaging the CPA and billing agreements may be cost-effective [34]. Cell therapies such as HSCT programs typically have the highest drug budgets and care acuity within healthcare institutions. Pharmacists contribute to cost control by streamlining the use of high-cost medications, implementing medication use algorithms, and prescription management, further saving pharmacists and physicians time to provide higher-value clinical services for more patients [34].

The inadequate and uncertainty of CAR T-cell therapy reimbursement persist. In August 2018, the U.S. Centers for Medicare & Medicaid Services (CMS) released the final rules for the FY 2019 Inpatient Prospective Payment System, which highlighted structural changes in new technology add-on payments for drug therapies and the creation of a new Medicare Severity Diagnosis-Related Group specifically for CAR T-cell therapy. Further complexities related to billing and reimbursement of CAR T-cell therapy also present an opportunity for the pharmacist to have a financial impact on cell collection, processing, infusion, and supportive care needed for this treatment modality [54].

Pharmacists provide personalized education and consultation to the patient, and their involvement in assessing and verifying the patient’s medication information makes the patient highly satisfied with such services [25, 29]. Furthermore, as one of the important healthcare providers, pharmacists can work collaboratively with multidisciplinary teams to reduce the workload of physicians, thereby minimizing burnout and enhancing their satisfaction [25]. The patient outcomes, considered as the most important measure of value, are influenced by multiple confounding factors, including the type of disease the patient has, the presence of other diseases before treatment, and variations in pharmaceutical care due to different treatment management plans.

When conducting interventions, pharmacists should consider controlling the variables in the process of medication management for patients, including medication reconciliation, medication and transplant education, medication therapy preparation, immunosuppression management, prophylactic medication management, Graft-versus-host disease and infection surveillance, and post-transplantation vaccine compliance [29]. Pharmacists adjust treatment plans in response to changes in patients’ clinical conditions, while tracking follow-up visits and time spent with patients to transform these services into measurable patient outcome metrics to deliver high-value care.

### Strengthen adaptation and development of prescribed practice standards

Future efforts should shift towards the standardization management of CGT/ATMPs, reflecting a scientifically sound and reasonable balance between safety and practicality, to ensure widespread access to these potentially transformative therapies or medical products [28, 61]. The adoption of existing clinical practice guidelines and procedures is an effective way to improve the standardization and quality of pharmaceutical practice. Some existing statements providing pharmacy practice management recommendations from the international pharmacy organizations were mentioned in the included studies, which were HCT clinical pharmacist role description statement (ASBMT pharmacy SIG) [62], recommendations for the role and competencies of the EBMT CP/P involved in HSCT (EBMT Pharmacist Committee) [40]. Pharmacy practice management and clinical management for COVID-19 in HSCT and cellular therapy patients (ASTCT Pharmacy SIG) [43] and guidance on the Pharmacy handling of Gene Medicines licensed GTMPs (European Association of Hospital Pharmacists) [63] were regarded as consensus recommendations for directing the design of questionnaires in the intervention studies [45, 54]. The full description of the above statements has been listed and categorized in Appendix table [40, 43, 62, 63]. The core roles and competencies of pharmacists listed therein, as well as specific pharmacy activities, could be used as learning materials for pharmacists to enhance their practical skills and understanding of specialist pharmacy services involved in participating the management of CGT/ATMPs. Professional recommendations or related consensus, critically reviewed and discussed by multiple stakeholders, also provide a structured reference for healthcare organizations seeking to implement or standardize pharmaceutical care processes. This alignment with internationally recognized standards helps promote the safe and effective management of such advanced therapies.

Beyond formal adoption of the existing guidance described previously, pharmacists could support best practices and provide the necessary clinical expertise and management of medications while participating the development of guidance and procedures in the institutional and committees to promote the quality and process improvement [64]. Prescription management activities are essential for the safe and effective use of medications and the achievement of reasonable cost control [37]. Pharmacists’ knowledge of complex formulation standards for sterile products or biologics, standards for hazardous medication, drug storage and delivery assessment, compliance with risk strategy plans, third-party payer rules/restrictions, health technology analysis, and clinical decision support strategies are all examples of the contribution of the pharmacist on such committees in the hospital setting. Pharmacists are also well-suited to participate in developing organizational patient care guidelines that incorporate evidence-based medicine and best medication practice. A well-informed example is the Pan UK ATMP Pharmacy Working Group (PWG) for ATMPs, which acts as an expert and informed body to support the activities of UK Pharmacies to facilitate ATMP usage. This group consists of pharmacists from across the UK that specialize in the governance, prescribing, administration and monitoring of ATMPs and is an excellent example of collaboration across the National Health Service (NHS) [65]. Many guidance reports issued by the PWG were covering the aspects of operational [66], clinical trials [67, 68] and regulatory [69, 70].

### Limitation

This study has some limitations. The range of designs and methodological approaches used by the studies included in this review—e.g., mixed methods, pre-post cohort intervention studies, retrospective studies, review, position statements, made it challenging to compare the data extracted from these studies. Furthermore, since the quality and bias risk of the included studies for this heterogeneity have not been assessed, it is necessary to interpret with caution to some extent. While this scoping review provides valuable insights, it is important to recognize that it is an indirect method of achieving the objectives. Adopting a more direct approach to studying the role of pharmacists in CGT/ATMPs interventions would provide a more comprehensive understanding of the current pharmacist's involvement in the management of such areas.

Despite the above-mentioned limitations, it is believed that this scoping review provided a comprehensive insight into a growing body of important literature that demonstrates the beneficial role of hospital pharmacists in the management of CGT/ATMPs.

## Conclusion

Many perceptions and findings from literature have demonstrated the positive role of pharmacists in the management of CGT/ATMPs, but there is currently limited evidence of efficacy for pharmacist-led interventions on patient outcomes. Leveraging the role of hospital pharmacists in multidisciplinary healthcare teams to develop a coordinated approach that supports pharmacy practice will better meet the management needs of CGT/ATMPs. Additionally, by continuing to enhance their advanced skillsets, focusing on the latest prescribed practice standards, and driving quality and process improvements, pharmacists will be better equipped to ensure the safe use of medications and enhance the quality of patient care.

## Supplementary Information

Supplementary Material 1.

Supplementary Material 2.

## Data Availability

No datasets were generated or analysed during the current study.

## Abbreviations

ATMPs: Advanced therapy medicinal products
ASBMT: American Society for Transplantation and Cellular Therapy
CAR: Chimeric antigen receptor
CRES: CAR T-cell-related encephalopathy syndrome
CGT: Cell and gene therapy
CPA: Collaborative practice agreement
CP/Ps: Clinical pharmacists/pharmacologists
CRS: Cytokine release syndrome
DRPs: Drug related problems
EBMT: European Society for Blood and Marrow Transplantation
EU: European Union
HSCT: Hematopoietic stem cell transplant
MTM: Medication therapy management
NHS: National Health Service
REMS: Risk Evaluation and Mitigation Strategy
SIG: Special Interest Group
SOP: Standard operating procedures
TDM: Therapeutic drug monitoring
USA: The United States of America
