# Supplementary material for: The role of hospital pharmacists in supporting the appropriate and safe use of CGT/ATMPs: a scoping review of current insights
Source: BMC Health Serv Res. 2025 Jan 9;25:52. doi: 10.1186/s12913-024-12026-4 (PMC11721208; doi:10.1186/s12913-024-12026-4)
Supplement: Supplementary file 1 — Supplementary Material 1. [file 12913_2024_12026_MOESM1_ESM.docx]

Appendix

Appendix. The description of pharmacists’ role/competency and pharmacy activities in the management of CGT/ATMPs in the involved statements

| **Statements/consensus (Issued institutions)** | **Target object** | **Role and core competencies recommended** | **Pharmacist Activities** | |
| --- | --- | --- | --- | --- |
| HCT clinical pharmacist role description statement (issued by ASBMT pharmacy SIG) [62] | HSCT patients | **Pharmacist’s core competencies**   - Medication management, including specialized knowledge of high-dose antineoplastics and infectious disease - Chemotherapy and medication counseling - Symptom management - Therapeutic drug monitoring - Discharge planning and transitions of care - Policy and guideline development - Education of team members, trainees, patients and families - Evidence-based program development and evaluation | **HCT Clinical Pharmacist Activities:**   1. Medication management and monitoring  - Provide thorough patient medication profile review - Participate in interdisciplinary rounding - Manage chemotherapy processes: Assistance with treatment planning and review or preparation of order set and policy documents - Assist with therapeutic drug monitoring: Immunosuppressants, antiinfectives, anti-seizure medications, anticoagulants, chemotherapeutic agents (e.g. busulfan), etc. - Provide medication therapy management: Diabetes, hypertension, dyslipidemia, etc. - Manage anti-infective therapies and promote stewardship: Prophylaxis and treatment recommendations; Monitoring  1. Patient Care  - Assist in symptom management: Supportive care; Pain/palliative care - Optimize graft-versus-host disease management - Facilitate post-transplant vaccine administration  1. Transition Planning  - Assist with transitions of care: Provide medication reconciliation; Collaborate on discharge planning and management - Facilitate transitions to hospice/palliative care where appropriate - Facilitate access to medications available through patient assistance programs  1. Education  - Patients and caregivers - Health care providers and trainees  1. Research and Quality Improvement  - Contribute to institutional and collaborative research and scholarly activities - Assist with policy and guideline development - Serve as patient and professional advocate - Monitor, evaluate, and report transplantrelated outcomes to assist in improvements to clinical practice | |
| Recommendations for the role and competencies of the EBMT CP/P involved in HSCT (issued by EBMT Pharmacist Committee) [40] | HSCT patients | **Recommendations for the role and competencies** | **Pharmacist activities** | |
|  |  | Patient care | *Mandatory activities* |  |
|  |  |  | - Pretransplant workup and verification of conditioning regimens (check the doses with respect to the protocol and the appropriate pharmaceutical stability in collaboration with the pharmacycompounding department) |  |
|  |  |  | - Medication reconciliation at the time of admission |  |
|  |  |  | - Assessment of current and past medical information/history, for example, pre-existing organ toxicity disorders that may have an effect on PK/PD (e.g., edema in the case of renal impairment) or pose a contraindication for some treatments |  |
|  |  |  | - Medication review to assess the appropriateness of the current medication (including allergies or possible drug interactions, use of herbal medicines) |  |
|  |  |  | - Identify and solve drug-related problems in the multidisciplinary team |  |
|  |  |  | - Documentation of drug-related problems and pharmacist interventions |  |
|  |  |  | - Prospective medication management (evaluation of appropriate indications, effectiveness, drug interactions, dosages, comorbidities, dose adjustments in patients with renal impairment, overweight/obesity or liver impairment…) |  |
|  |  |  | - Consideration of patient preferences, e.g., pharmaceutical formulations, - especially in pediatric or elderly |  |
|  |  |  | - Identification, reporting, recording, and preventing of adverse drug - events and medication errors |  |
|  |  |  | - Participation in multidisciplinary meetings and ward rounds |  |
|  |  |  | - Medication reconciliation at the time of discharge |  |
|  |  |  | - Therapeutic drug monitoring (TDM) (drugs with a narrow therapeutic window: chemotherapy, immunosuppressives, antiepileptics, antiinfectives…) (performing and/or supervising) |  |
|  |  |  | *Optional activities* |  |
|  |  |  | - Analysis of financial issues (reimbursement of drugs) and facilitation of access to drugs |  |
|  |  |  | - Nutrition support (enteral and parenteral) |  |
|  |  | Hospital pharmacy services | *Mandatory activities* |  |
|  |  |  | - Preparation of cytotoxics |  |
|  |  |  | - Drug information service |  |
|  |  |  | - Computerized Physician Order Entry with Clinical Decision Support System |  |
|  |  |  | - Advanced therapy medicinal products (for sites using ATMPs) - To liaise with pharmacy colleagues to undertake a feasibility assessment for the use of the ATMP - To ensure that hospital governance processes for ATMPs have been documented and followed - To document a procedure for the use of the ATMP detailing ordering receipt, storage, preparation, administration, and monitoring/follow-up, stating responsibilities of the multidisciplines involved - To assess any further pharmacy capacity implications as a result of potential toxicities, e.g., preparation of monoclonal antibodies, PN, or intrathecal injections - To liaise with clinical trials and research colleagues, where the ATMP is also an investigational medicinal product, to ensure GCP compliance |  |
|  |  |  | *Optional activities* |  |
|  |  |  | - Unit-dose drug distribution |  |
|  |  | Process of care | *Mandatory activities* |  |
|  |  |  | - Development of guidelines and standard operating procedures related to HSCT and supportive care |  |
|  |  |  | - Assist in decision-making for hospital formularies for HSCT patients in collaboration with other members of the Medical Pharmaceutical Committee |  |
|  |  |  | *Optional activities* |  |
|  |  |  | - Drug use evaluations (drugs and medical devices) |  |
|  |  | Research | *Optional activities* |  |
|  |  |  | - PK–PD modeling for individualized dosing of drugs used in HSCT - Population–pharmacokinetic/pharmacodynamic modeling - Identifying optimal exposures for most drugs in HSCT - Tailored dosing for optimal outcome, can be used with or without TDM - Comparing different exposure measures, with the goal of setting a uniform target for individualized dosing and/or TDM - Development and implementation of TDM - Developing robust and fast assays for TDM - Investigating the added value of TDM to patient outcomes and comparing different TDM strategies - Clinical research and support in clinical trials - Available for consultation in any drug-related trial within EBMT, especially in terms of pharmacokinetics and of pharmacodynamics - Development of a central database for centers of expertise for druglevel quantification |  |
|  |  | Education and training | *Mandatory activities* |  |
|  |  |  | - Patient education (pretransplant visit, at the time of transition: admission and discharge) - Staff education (physicians, nurses, pharmacists, and pharmacist technicians)—possible topics: - Supportive care, e.g., anti-infectives, cytotoxics, anti-emetics, or nutrition ○Safe handling of cytotoxics - Compatibility of drugs - Drug administration via feeding tubes - Central venous catheter, e.g., choice, maintenance procedures, or infusion lines - Compounding of cytotoxics - TDM - Handling of cellular medicines (ATMPs) |  |
|  |  |  | *Optional activities* |  |
|  |  |  | - Student education |  |
|  |  | Quality and process improvement | *Mandatory activities* |  |
|  |  |  | - JACIE accreditation requirements for pharmacists (according to the latest JACIE Standards Edition) |  |
|  |  | Pharmacoeconomics | *Mandatory activities* |  |
|  |  |  | - Involvement in decision-making of drug formularies |  |
|  |  |  | - Involvement in cost reduction or improved reimbursement strategies |  |
|  |  |  | *Optional activities* |  |
|  |  |  | - Pharmacoeconomic analyses |  |
| Pharmacy practice management and clinical management for COVID-19 in HSCT and cellular therapy patients (issued by ASBMT pharmacy SIG) [43] | HSCT and cellular therapy patients in the COVID-19 settings | **Pharmacist’s core roles for inpatient**  Limiting exposure to staff and patients | **Inpatient Considerations for Pharmacy Practice Management**  Limiting physical presence   - Attending clinical rounds or huddles virtually - Rotating pharmacy staff on- and off-site - Pharmacists to avoid accessing patient rooms (not seeing patients directly) - If rounding in person, maintaining 6 feet from other team members | |
|  |  | Distribution of services Onsite | Onsite   - Urgent/emergent needs (rapid responses/codes) - Collaboration with other team members - Discharge education   Offsite   - Medication education and reconciliation - Patient own medication identification - Therapeutic drug monitoring - Order verification, including chemotherapy - Medication adjustments based on renal and liver function - Drug-drug interaction management | |
|  |  | Technology | Messaging   - Chat mechanisms (EMR real-time, Cureatr, Voatle, Skype for Business) - EMR messaging functions - Email - Handouts or medication calendars sent to patient via EMR or through programs such as MedActionPlanPro   Audiovisual   - Audio call resources (Doximity, Jabber phone, blocked calls from personal line) - Virtual visit capabilities (eg, webcam) - Zoom/Webex/Skype for Business (for communicating with team members) (if approved for use by IT department) | |
|  |  | **pharmacist’s core roles for outpatient** | **Outpatient Considerations for Pharmacy Practice Management** | |
|  |  | Limiting exposure to staff and patients | Limiting physical presence   - Concentrating visits on particular days to allow for non-patient visit days - Rotating pharmacy staff on- and off-site - Pharmacists to avoid accessing patient rooms (not seeing patients directly) | |
|  |  | Distribution of services | Onsite   - Urgent/emergent needs (rapid responses/codes) - Help maintain clinic workflow efficiency/throughput - Collaboration with other team members   Offsite   - Medication education and reconciliation - Oral chemotherapy education and follow-up - Conditioning and transplant education - Laboratory follow up - Therapeutic drug monitoring - Order verification - Chemotherapy order preparation | |
|  |  | Technology | Messaging   - Chat mechanisms (EMR real-time, Cureatr, Voatle, Skype for Business) - EMR messaging functions - Email - Handouts or medication calendars sent to patient via EMR or through programs such as MedActionPlanPro   Audiovisual   - Audio call resources (Doximity, Jabber phone, blocked calls from personal line) - Virtual visit capabilities (eg, webcam) - Zoom/Webex/Skype for Business (for communicating with team members) (if approved for use by IT department) | |
| Guidance on the Pharmacy handling of Gene Medicines licensed GTMPs (issued by European Association of Hospital Pharmacists) [63] | Gene medicines licensed GTMPs | **Pharmacist’s core competency**  Handling of gene medicines and associated patient specimens | **Guidance for handling of gene medicines and associated patient specimens**   - Universal precautions must be observed in the handling of gene medicines, clinical specimens (patient blood, tissue, body fluids) and materials or equipment contaminated by these substances following treatment. - Wear suitable protective clothing to minimise the risk of microbiological contamination of the therapeutic agent during preparation. The clothing (see right-hand box) and its quality should be appropriate to protect the therapeutic agent from contamination - Use of a biological safety cabinet or pharmaceutical grade isolator (compliant with European standard EN 12469:2000) – dispensation and preparation of gene medicines (minimum class II, type B) - Needles and sharps: take adequate precaution with use and disposal - Ensure decontamination of work surface areas - Clean/decontaminate patient bedding according to procedures used for blood- or body-fluid-soiled laundry* - No special precautions for patient elimination of stools or urine, unless specified in the SPC for product-specific information - Transport and storage of patient specimens must be in a closed, labelled leak-proof container - Disposal of products, contaminated waste (e.g. gloves, gowns, etc.) and patient specimens must follow the local procedures of the institution for decontamination   *Disposable laundry is preferred unless there is solid evidence that the vector is not being shed. | |
